# Supplementary material for: A metastable liquid melted from a crystalline solid under decompression
Source: Nat Commun. 2017 Jan 23;8:14260. doi: 10.1038/ncomms14260 (PMC5264249; doi:10.1038/ncomms14260)
Supplement: Supplementary Information — Supplementary Figures, Supplementary Tables and Supplementary References [file ncomms14260-s1.pdf]

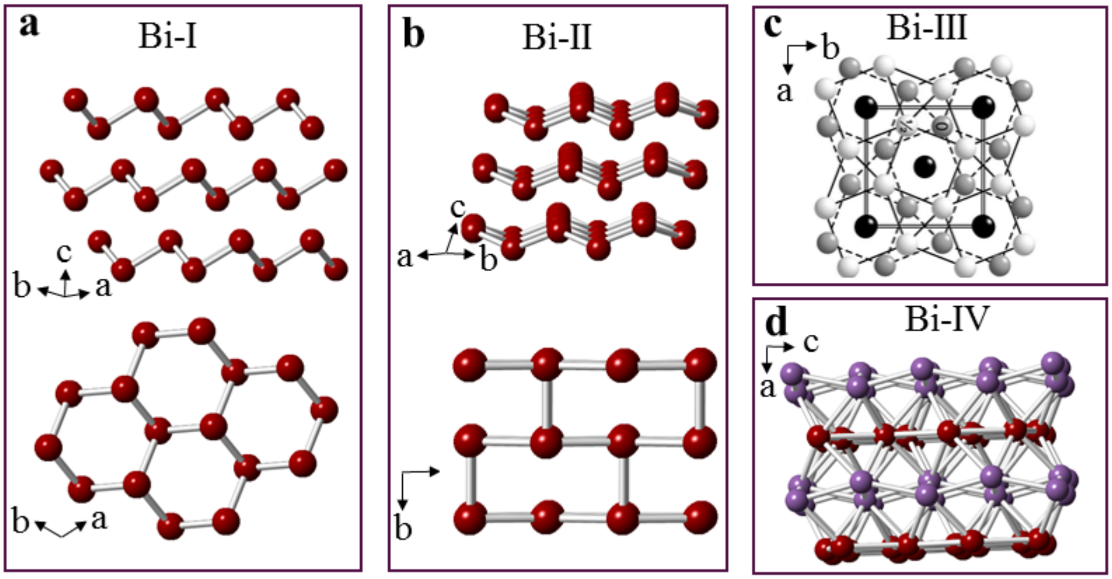

Supplementary Figure 1 | Crystal structures of different Bi phases. (**a**) Structure of Bi-I[1]. (**b**) Structure of Bi-II[2]. (**c**) Structure of Bi-III[3]. (**d**) Structure of Bi-IV[4].

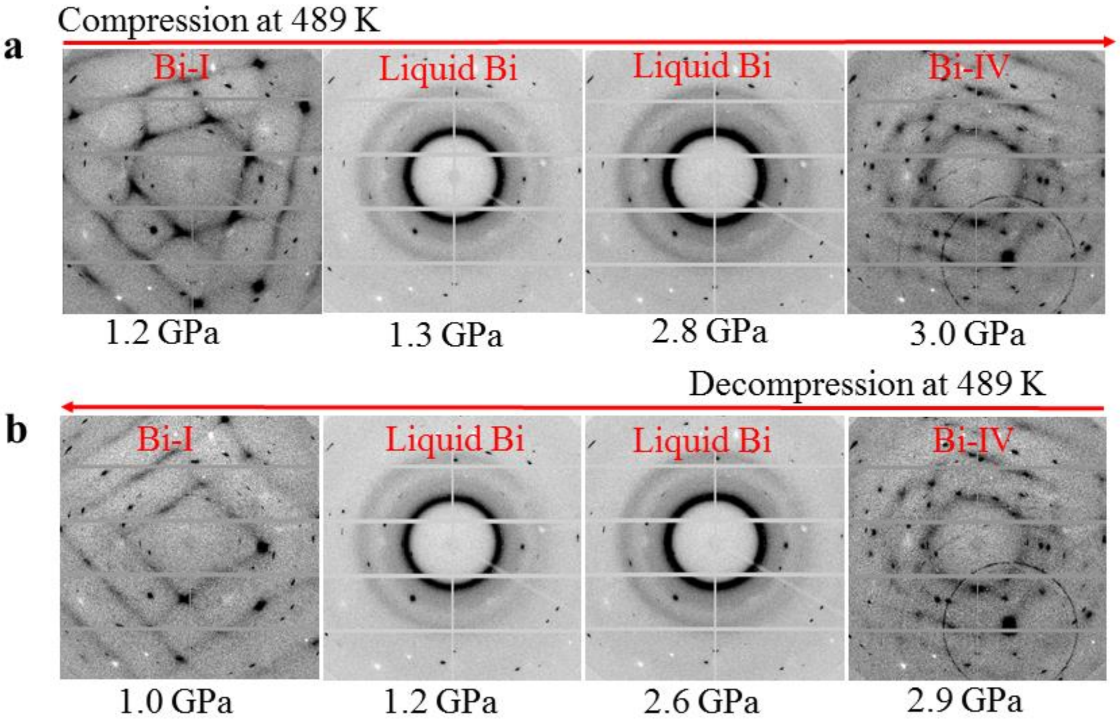

Supplementary Figure 2 | Diffraction images at 489 K with NaCl as pressure medium. (**a**) Sample Bi was compressed from 0.5 GPa to 3.2 GPa (Right red arrow). Bi-I phase transforms to liquid Bi at ~1.3 GPa, followed by crystallization into Bi-IV at ~2.8 GPa. (**b**) Sample Bi is decompressed from 3.2 GPa to 0.5 GPa (left red arrow). Bi-IV melts into liquid Bi at ~2.6 GPa, followed by crystallization into Bi-I at ~1.2 GPa. The background is subtracted for all images. The diffraction spots in liquid Bi come from the pressure medium of NaCl or the diamond anvils.

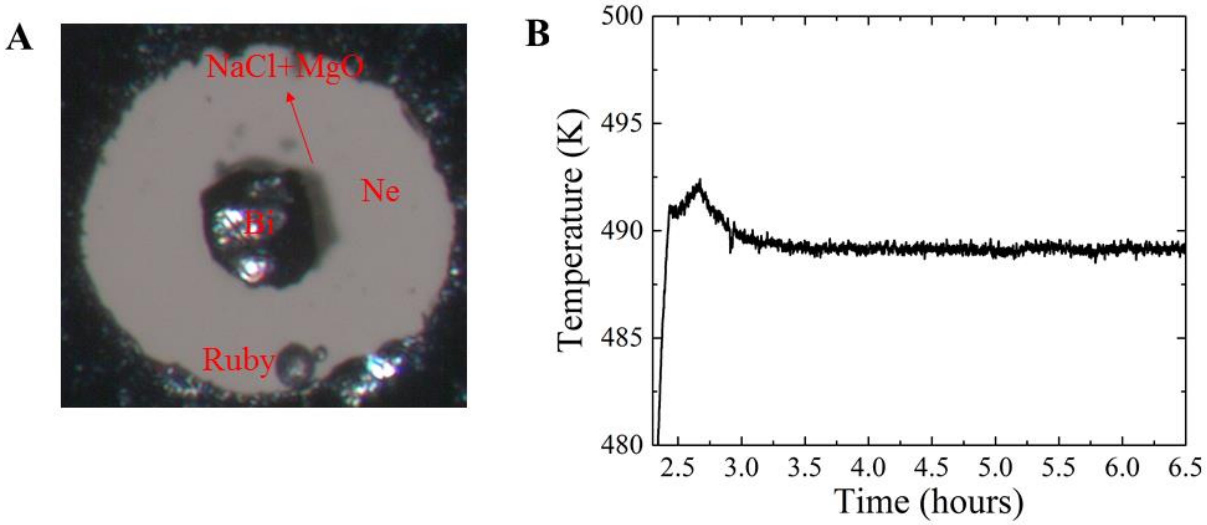

Supplementary Figure 3 | Photo of a sample chamber after gas loading and typical temperature variations in experiments. (**a**) A photo of a sample chamber at 0.5 GPa after neon was loaded. The sample chamber is ~150 μm in diameter, and the bismuth sample dimension is 30×30 μm. (**b**) Typical temperature variations recorded as a function of time during one experiment. The Bi sample is compressed and decompressed after temperature becomes stable within a degree.

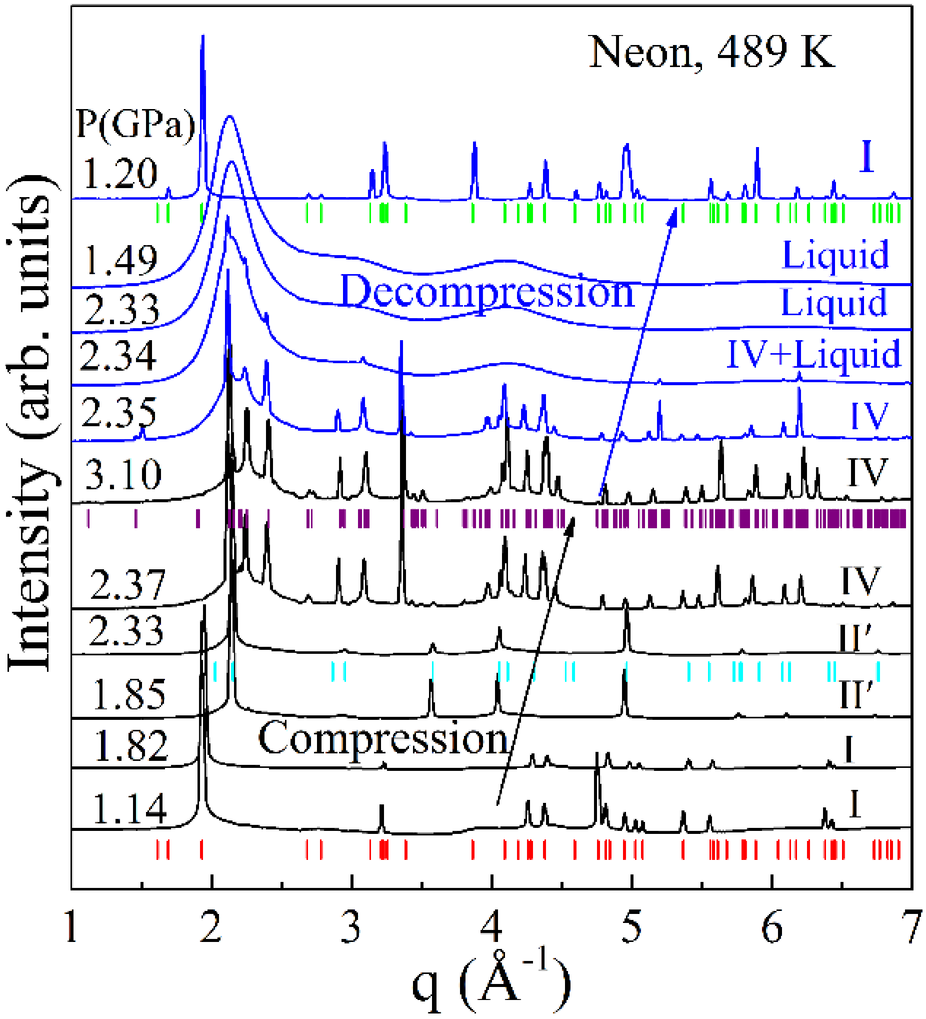

Supplementary Figure 4 | Integrated x-ray diffraction patterns of Bi at 489 K. The Bi sample is compressed and decompressed at hydrostatic condition with neon as pressure medium. Upon compression (black arrow). Bi-I phase transforms to Bi-II´ at ~1.8 GPa, followed by II´-to-IV transition at ~2.4 GPa. Blue arrow indicates that Bi is decompressed from 3.1 GPa to 1.0 GPa. Bi-IV melts into liquid Bi at ~2.3 GPa, followed by crystallization of liquid Bi at ~1.2 GPa. The symbol bars below each of the corresponding diffraction patterns indicate the indexing of the crystal phases.

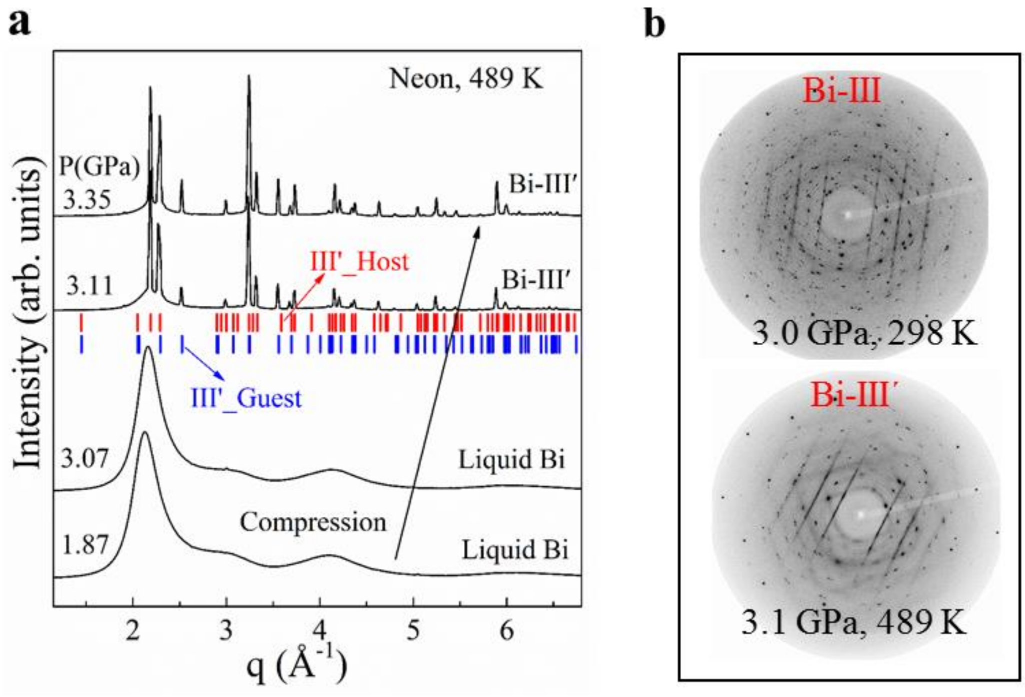

Supplementary Figure 5 | Phase transition of DIL under hydrostatic condition at 489 K. Neon is used as pressure medium. (**a**) The DIL is compressed from ~1.9 GPa to 3.3 GPa. It crystallizes into Bi-III-like phase (Bi-III´) at ~3.1GPa, which can be indexed using a mixture of two body-centered-tetragonal phases (red and blue bars). The lattice parameters are close to those of the host and guest structures of Bi-III at ~3 GPa[1]. (**b**) Comparison of diffraction images of Bi-III´ and Bi-III. There are similar straight diffuse scattering lines in both Bi-III and Bi-III´, which comes from the disordered guest chains[5-9].

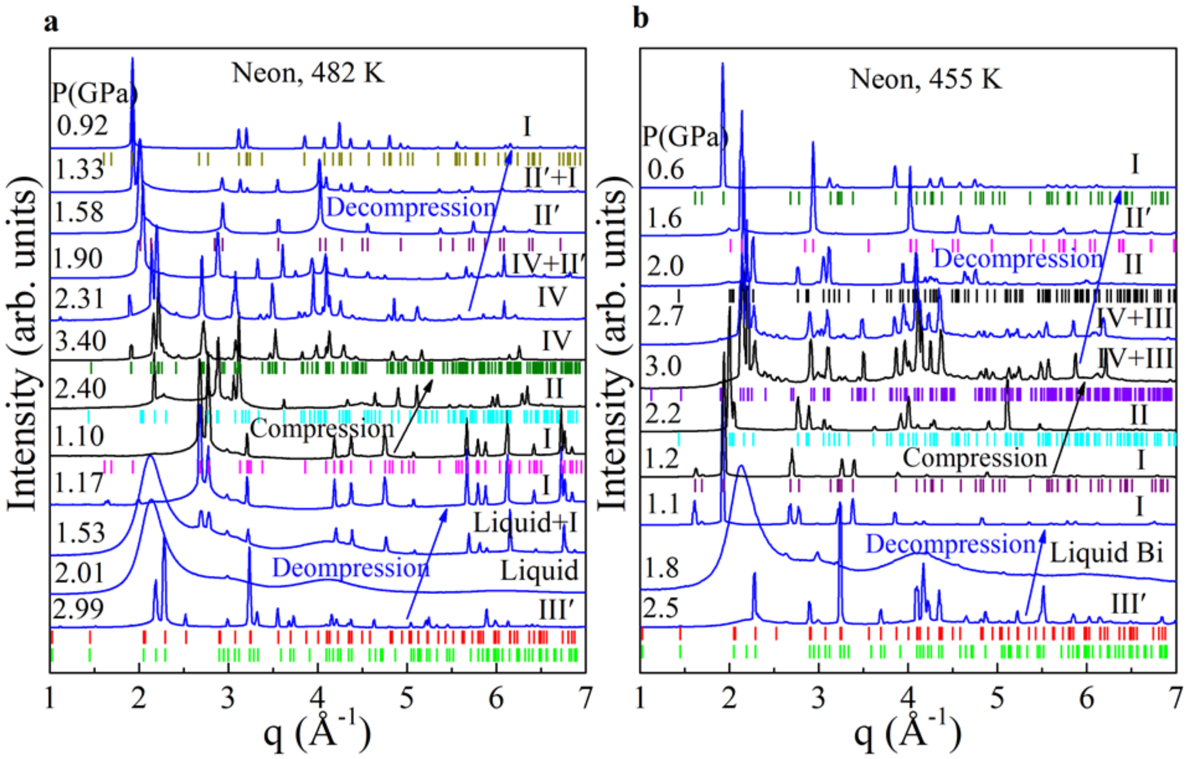

Supplementary Figure 6 | Integrated x-ray diffraction patterns of Bi under hydrostatic condition. Neon is used as pressure transmitting medium. (**a**) Sample is decompressed from ~3.0 GPa to 1.1 GPa (blue arrow) at 482 K. III′→ liquid→ I transitions are observed. Then sample is compressed from 1.1 GPa to 3.4 GPa with I→ II→ IV transitions (black arrow). When the starting phase is IV, IV→ II′→ I transitions appear (blue arrows on the top). (**b**) Sample is decompressed and compressed at 455 K. When decompressing Bi-III′, III′→ liquid→ I transitions are observed while decompressing mixture of IV+III led to IV+III→ II→ I transitions. Under compression, I→ II→ IV transitions appear. The symbol bars below the corresponding diffraction patterns indicate the indexing of the crystal phases.

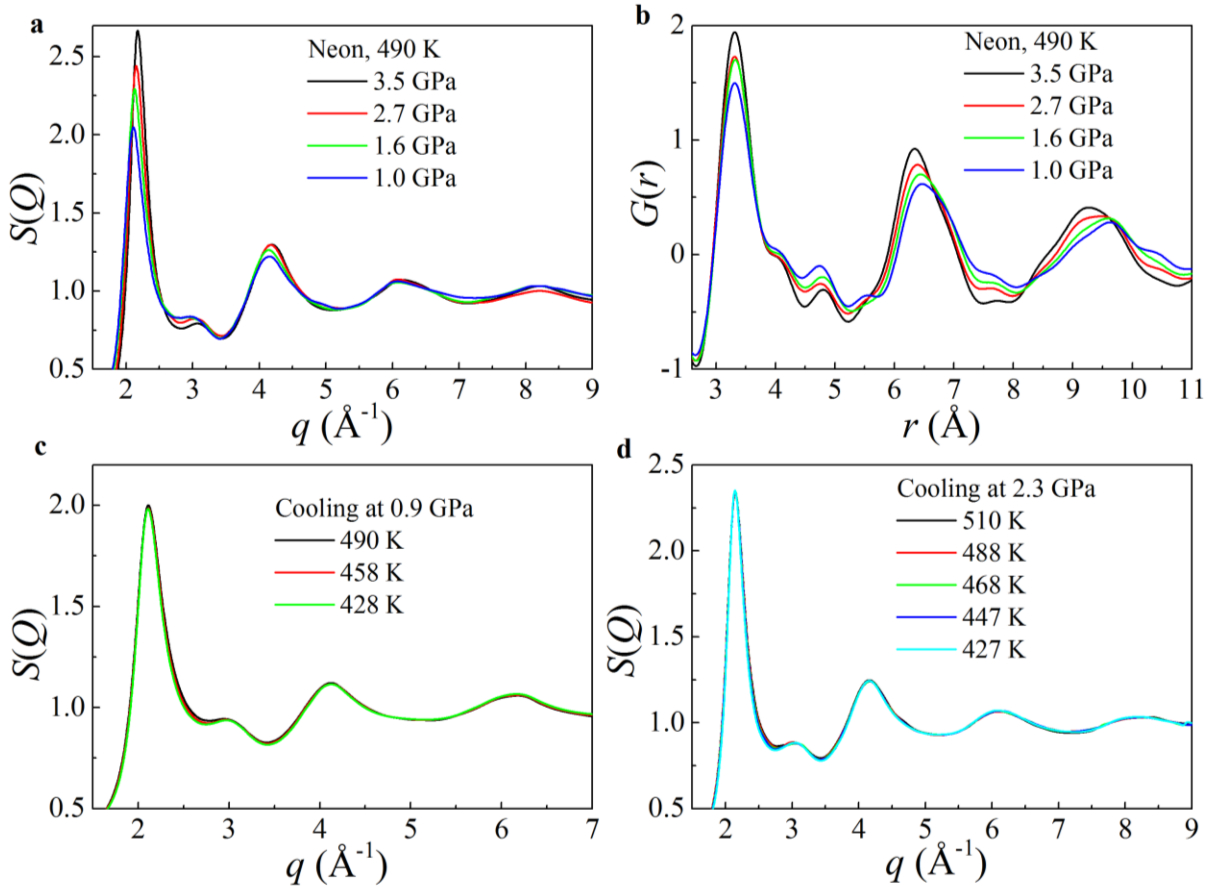

Supplementary Figure 7 | Effect of temperature and pressure on the structure of DIL and supercooled liquid. (**a**) Evolution of structure factors, S(*Q*), of the DIL under various pressures at 490 K. (**b**) Reduced pair distribution functions, *G*(*r*) of the DIL under pressures. (**c**) *S*(*Q*) of the supercooled liquid Bi at 0.9 GPa. (**d**) *S*(*Q*) of the supercooled liquid Bi at 2.3 GPa.

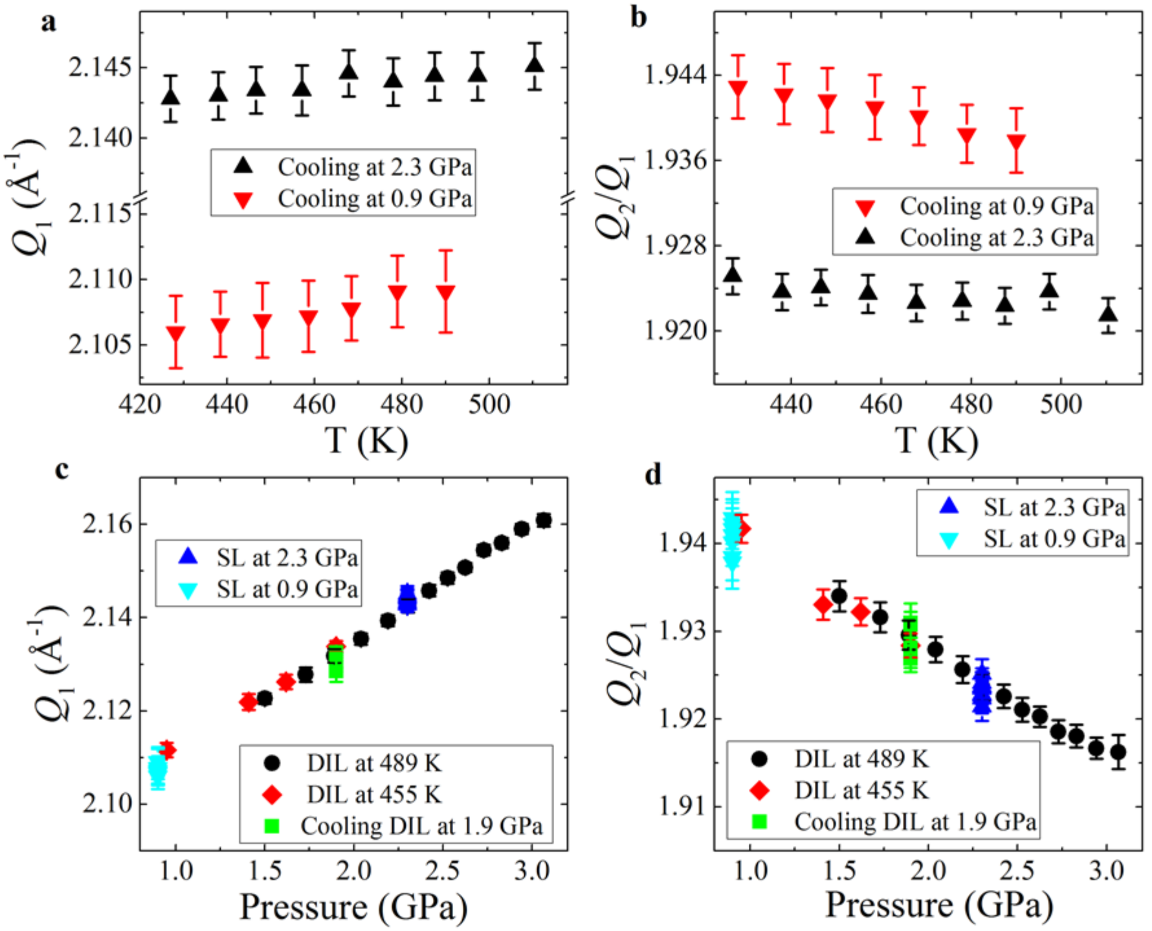

Supplementary Figure 8 | Pressure and temperature dependences of Q_1 and Q_2/Q_1 of the DIL and the supercooled liquid. (**a**) and (**b**) Temperature-dependence of Q_1 and Q_2/Q_1 under cooling at 0.9 GPa and 2.3 GPa, respectively. Q_1 decreases slightly during cooling while Q_2/Q_1 increases slightly with cooling. (**c**) and (**d**) Pressure-dependences of Q_1 and Q_2/Q_1 of the DIL. When the data of supercooled liquid Bi in (**a**) and (**b**) were over-plotted on (**c**) and (**d**), we find that Q_1 and Q_2 of the supercooled liquid at different temperatures are close to those of the DIL at corresponding pressures, indicating the structural similarity of the DIL with the supercooled liquid.

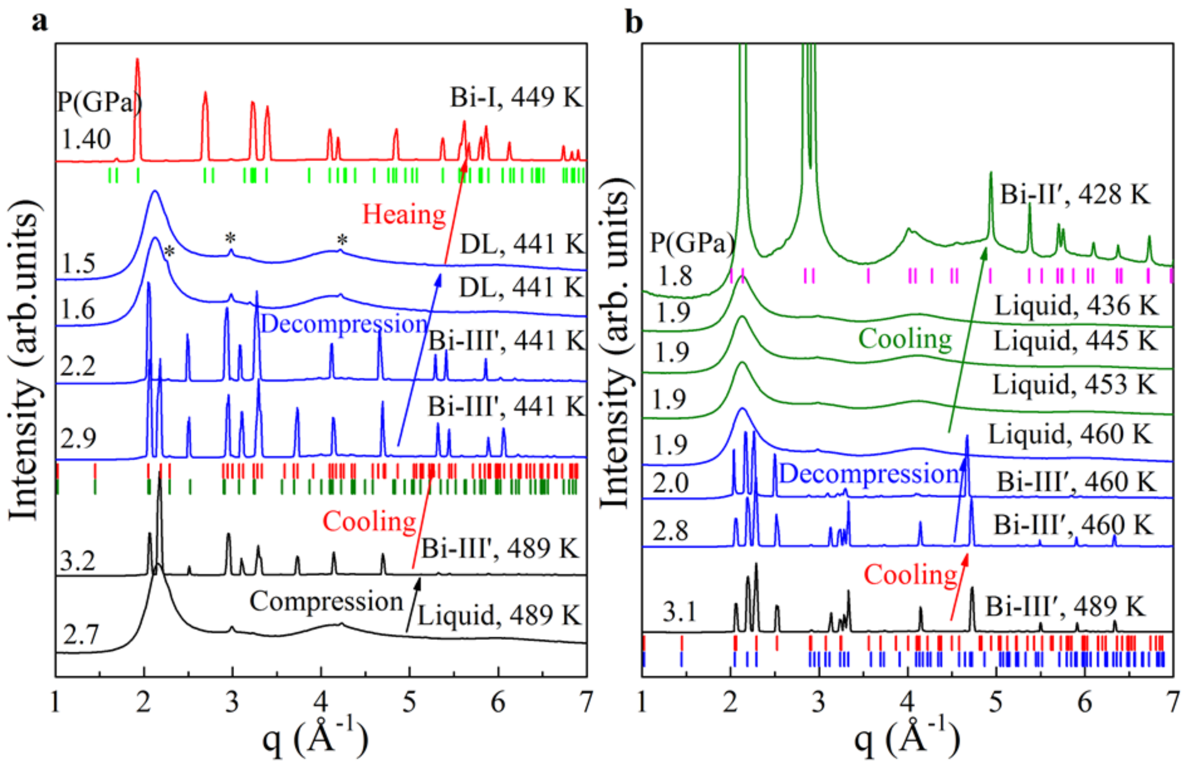

Supplementary Figure 9 | Crystallization of the DIL under heating and cooling. Bi-III´ is obtained by compressing liquid Bi above 489 K. (**a**) The DIL is compressed at 489 K and transformed to Bi-III´. Then Bi-III´ is cooled from 489 K to 441 K. Keeping the temperature at 441 K, Bi-III´ is decompressed and melts into a liquid state under decompression. After DIL is obtained at 441 K, the metastable liquid Bi is heated from 441 K to 449 K and crystallizes into Bi-I during heating. After crystallization, the pressure changes from 1.5 GPa to 1.4 GPa. (**b**) Decompression-induced liquid crystallizes into Bi-II under cooling from 460 K to 428 K. The pressure changes from 1.9 GPa to 1.8 GPa after crystallization.

Supplementary Table 1 | Unit cell parameters of Bi-IV, III´, II, II´ and I. The structural models of these crystalline phases are from Refs. 1, 2, 4, 10.

	**IV (2.97 GPa)**	**III´ (3.46 GPa)**		**II (2.0 GPa)**	**II´ (2.0 GPa)**	**I (1.14 GPa)**
Structure	Orthorhombic (*Cmca*)	Tetragonal (host: *I4/mcm*)	Tetragonal (Guest: *I4/mmm*)	Monoclinic (*C2/m*)	Tetragonal	Hexagonal
a	11.178(1) Å	8.673(3) Å	8.670(3) Å	6.663(4) Å	6.222(3) Å	4.522(1) Å
b	6.618(1) Å			6.181(3) Å		
c	6.596(1) Å	4.266(5) Å	3.247(3) Å	3.312(1) Å	3.320(1) Å	11.681(3) Å
β				110.93(2)°		
V	487.92 (9) Å^3	320.9(3) Å3	244.1(2) Å^3	127.39(5) Å^3	128.5(1) Å^3	206.90(5) Å^3

Supplementary Table 2 Summary of the phase transitions under hydrostatic condition and different temperatures. Red text indicates the occurrence of decompression-induced liquid, i.e., crystal-liquid-crystal transitions, while crystal-crystal-crystal transitions are observed under compression. The last column indicates the numbers of repeated experiments at corresponding temperatures. In each experimental run, a fresh sample is used.

**T (K)**	**Compression**	**Decompression**	**Times**
505	I → Liquid → Bi-N	IV → Liquid → I	1
489	I → II′→ IV	IV→ Liquid→ I	5
	Liquid→ III′	III′ → Liquid → I	5
482		III′ → Liquid→ I	1
	I→ II→ IV	IV → II′ → I	1
455		III′ → Liquid → I	2
	I→ II→ IV+III	IV+III → II→ II′ →I	2
441	I→ II→ III	III′ → Liquid → I	1
423	I→ II→ III	III′ → II → I	2

SUPPLEMENTARY REFERENCES:

1. Katzke, H. & Toledano, P. Displacive mechanisms and order-parameter symmetries for the A7-incommensurate-bcc sequences of high-pressure reconstructive phase transitions in Group Va elements. *Phys. Rev. B* **77**, 024109 (2008).
2. Akselrud, L. G., Hanfland, M. & Schwarz, U. Refinement of the crystal structure of Bi-II, at 2.54 GPa. *Z Krist-New Cryst. St.* **218**, 415-416 (2003).
3. McMahon, M. I., Degtyareva, O. & Nelmes, R. J. Ba-IV-type incommensurate crystal structure in group-V metals. *Phys. Rev. Lett.* **85**, 4896-4899 (2000).
4. Chaimayo, W. *et al.* High-pressure, high-temperature single-crystal study of Bi-IV. *High Pressure Res.* **32**, 442-449 (2012).
5. Nelmes, R. J., Allan, D. R., McMahon, M. I. & Belmonte, S. A. Self-hosting incommensurate structure of barium IV. *Phys. Rev. Lett.* 83, 4081-4084 (1999).
6. McMahon, M. I. & Nelmes, R. J. Chain "melting" in the composite Rb-IV structure. *Phys. Rev. Lett.* 93, 055501 (2004).
7. Falconi, S. et al. X-ray diffraction study of diffuse scattering in incommensurate rubidium-IV. *Phys. Rev. B* 73, 214102 (2006).
8. McBride, E. E. et al. One-dimensional chain melting in incommensurate potassium. *Phys. Rev. B* 91, 144111 (2015).
9. McMahon, M. I., Bovornratanaraks, T., Allan, D. R., Belmonte, S. A. & Nelmes, R. J. Observation of the incommensurate barium-IV structure in strontium phase V. *Phys. Rev. B* 61, 3135-3138 (2000).
10. Principi, E. *et al.* Metastable phase diagram of Bi probed by single-energy x-ray absorption detection and angular dispersive x-ray diffraction. *Phys. Rev. B* **74**, 064101 (2006).
